# 808-nm Photobiomodulation Affects the Viability of a Head and Neck Squamous Carcinoma Cellular Model, Acting on Energy Metabolism and Oxidative Stress Production

**DOI:** 10.3390/biomedicines9111717

**Published:** 2021-11-18

**Authors:** Silvia Ravera, Nadia Bertola, Claudio Pasquale, Silvia Bruno, Stefano Benedicenti, Sara Ferrando, Angelina Zekiy, Praveen Arany, Andrea Amaroli

**Affiliations:** 1 Department of Experimental Medicine, University of Genoa, 16132 Genoa, Italy; silvia.ravera@unige.it (S.R.); nadia.bertola@edu.unige.it (N.B.); silvia.bruno@unige.it (S.B.); 2 Department of Surgical and Diagnostic Sciences, University of Genoa, 16132 Genoa, Italy; clodent@gmail.com (C.P.); stefano.benedicenti@unige.it (S.B.); 3 Department of Earth, Environmental and Life Sciences, University of Genoa, 16132 Genoa, Italy; sara.ferrando@unige.it; 4 Department of Orthopedic Dentistry, Faculty of Dentistry, First Moscow State Medical University (Sechenov University), 119991 Moscow, Russia; zekiy82@bk.ru; 5 Departments of Oral Biology, Surgery and Biomedical Engineering, University at Buffalo, Buffalo, NY 14260, USA; prarany@buffalo.edu

**Keywords:** apoptosis, antioxidant defenses, light therapy, low-level laser therapy, oral cancer, oxidative phosphorylation, phototherapy, supportive care

## Abstract

Photobiomodulation (PBM) is a form of low-dose light therapy that acts through energy delivery from non-ionizing sources. During the recent two decades, there has been tremendous progress with PBM acceptance in medicine. However, PBM effects on potential stimulation of existing malignant or pre-malignant cells remain unknown. Thus, the primary endpoint was to assess the safety of PBM treatment parameters on head and neck squamous cell carcinoma (HNSCC) proliferation or survival. The secondary endpoint was to assess any putative anti-cancer effects of PBM treatments. Cell viability, energy metabolism, oxidative stress, and pro- and anti-apoptotic markers expression were investigated on a Human Head and Neck Squamous Cell Carcinoma cellular model (OHSU-974 FAcorr cell line). PBM therapy was administered through the 810 nm diode laser (GaAlAs) device (Garda Laser, 7024 Negrar, Verona, Italy) at the powers of 0, 0.25, 0.50, 0.75, 1.00, or 1.25 W in continuous wave (CW) mode for an exposure time of 60 s with a spot-size of 1 cm^2^ and with a distance of 1.86 cm from the cells. Results showed that 810-nm PBM affected oxidative phosphorylation in OHSU-971 FAcorr, causing a metabolic switch to anaerobic glycolysis. In addition, PBM reduced the catalase activity, determining an unbalance between oxidative stress production and the antioxidant defenses, which could stimulate the pro-apoptotic cellular pathways. Our data, at the parameters investigated, suggest the safeness of PBM as a supportive cancer therapy. Pre-clinical and clinical studies are necessary to confirm the in vitro evidence.

## 1. Introduction

Photobiomodulation (PBM) is a low-dose light therapy form, which affects cell metabolism through energy delivery from non-ionizing sources [1]. Photons in the visible and near-infrared spectrum can excite organic molecules, such as iron-sulfur and heme proteins, cytochromes, and porphyrins. These chromophores into the non-plant cells can receive and transform photonic illumination into biochemical energy [2]. The mitochondrial electron transport complexes, especially complex IV, play a pivotal role in visible and NIR light–cell interaction [3,4,5,6]. Additionally, the roles of water [7], lipids [8], transforming growth factor-β (TGF-β) [9], and voltage-gated calcium channels [10] have been noted as targets for light interactions. These first reactions stimulate a cascade of other pathways leading to up-regulation of ATP [3,4,5,6], modulation of calcium [1,10,11] and cytokine homeostasis [12], nitric oxide (NO) and reactive oxygen species (ROS) production [13,14], change in redox signaling [15], and release of growth factors [9,12]. The process results in promising therapeutic outcomes such as promotion of tissue repair and regeneration [16,17,18], reduction of pain [8], alleviation of neurological disorders [19,20,21], and immunomodulation [22].

During the recent two decades, there has been tremendous progress with PBM acceptance in medicine. Its potent efficacy has been noted in supportive cancer therapy for cancer treatment complications, such as oral mucositis (OM), radiodermatitis, lymphedema, and peripheral neuropathy [23,24,25,26,27,28]. The Mucositis Study Group of the Multinational Association of Supportive Care in Cancer/International Society for Oral Oncology (MASCC/ISOO) has unequivocally recommended the use of PBM therapy in the prevention of OM in Head and Neck Squamous Cell Carcinoma (HNSCC) patients on chemoradiotherapy [24,27]. This is particularly relevant as HNSCC is the seventh most common cancer worldwide, with over 700,000 new cases diagnosed yearly [29,30]. Moreover, the PBM treatments for the prevention of OM in HNSCC patients potentially expose tumor cells within the treatment field. There remain concerns on the unknown effects of PBM on potential stimulation of existing malignant or pre-malignant cells as well as induction of therapeutic resistance due to improved vascularity or modulation of immune responses [23,24,31].

Energy metabolism differs in cancer cells compared to healthy cells. Specifically, no-cancer cells are characterized by a high rate of aerobic metabolism. Conversely, cancer cells increase glucose consumption and lactic acid release, despite normoxic conditions, modifying the microenvironment [32]. This condition is known as the Warburg effect [33]. However, cancer cells also maintain the mitochondrial function to produce some of the energy required for their proliferation [34]. Regarding metabolic modulation, in vitro and in vivo research studies have shown contradictory effects of PBM on normal versus cancer cells [23,31,35]. For example, ATP generation and mitochondria activity modulation due to PBM treatments affect cancer cell pro-apoptotic cytotoxic stimuli, inducing energy-dependent cell death programs [31,36,37]. At the same time, activation of PI3K/AKT/mTOR, TGF-β, MAPK, and heat shock proteins pathways, which are generally associated with PBM effects on normal cells [26,31], generates concerns on the safety in cancer cells.

Thus, the potential *Janus Bifrons* relation between the clinical application of PBM and its effects on cancer cells has yet to be elucidated. Specifically, the PBM effect on mitochondria energy metabolism in carcinoma cells remain to be investigated [38]. Therefore, to increase PBM therapy acceptance and to elucidate the effect of PBM treatments on cancer cells, this study focused on examining the 808 nm wavelength treatments at increasing power from 0.25 Watt (W) up to 1.25 W for 60 s on a 1 cm^2^ area on an HNSCC cancer cell line, OHSU-974 FAcorr. The first endpoint here was to assess the safety of PBM treatment parameters on HNSCC proliferation or survival. A second endpoint was to investigate any putative anti-cancer effects of PBM treatments. Cell viability, metabolism, oxidative stress, and pro- and anti-apoptotic markers expression were investigated.

## 2. Materials and Methods

### 2.1. Experimental Design

**Figure 1:**
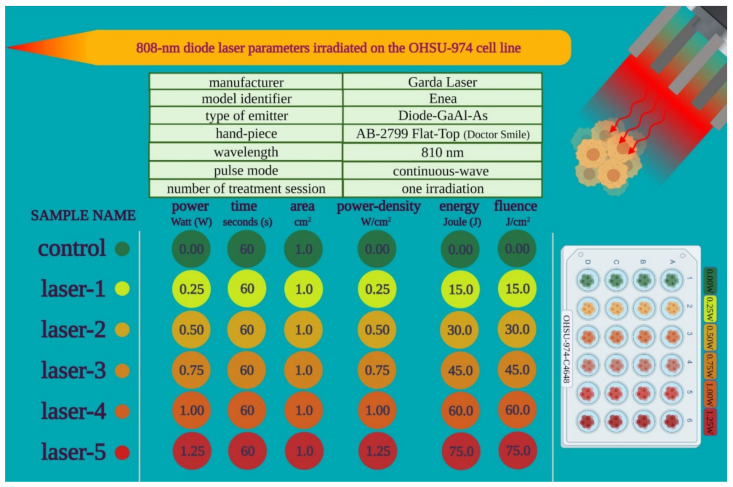
Outline of the PBM treatment parameters used in the study.

We performed PBM with an 810 nm diode laser (GaAlAs) device (Garda Laser, 7024 Negrar, Verona, Italy). The laser was equipped with the AB-2799 hand-piece irradiating through a flat-top beam profile (Doctor Smile, LAMBDA Spa, 36100 Vicenza, Italy). The flat-top hand-piece was chosen because, as we previously showed, it can generate a homogeneous power density on the beam spot-area, which is not affected by the distance from the target [36,37]. Therefore, the flat-top technology supports the uniformity of photobiomodulation treatments and enhances the rigor and reproducibility of PBM clinical outcomes [39,40]. PBM therapy parameters were chosen based on our prior work showing the supportive effect of 808 nm laser treatment at 1 W for 60 s with a spot size area of 1 cm^2^ in vitro on endothelial [41] and stem cells [42,43], as well as in vivo on patients affected by aphthae [44] and Bell’s palsy [45]. To support the safety of 808 nm PBM and in accordance with a possible power decrement across tissues, a range of powers from 0.25 W up to 1.25 W was examined, as described in Figure 1.

**Figure 2:**
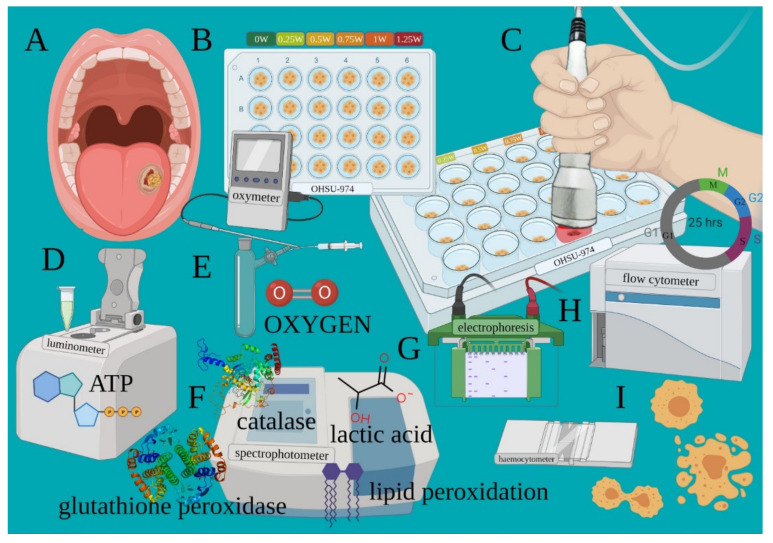
Experimental design outlining the various outcomes assessed after PBM treatments. Cancer cell line from human tongue squamous cell carcinoma (**A**) was transferred into a black 24 well plate with a flat and clear bottom (**B**). PBM therapy was administered through the 810-nm laser (**C**). After treatments, cells are processed to evaluate the variation of the energetic metabolism such as ATP synthesis (**D**), oxygen consumption (**E**), OxPhos efficiency, lactic acid production and oxidative stress (**F**). Markers regulating apoptosis (**G**) and cell cycle and viability (**H**,**I**) were also detected.

The cancer cell line from human tongue squamous cell carcinoma (Figure 2A) was transferred into a black 24 well plate with a flat and clear bottom (Figure 2B). Treatments were performed with the flat-top hand-piece in contact with the well top, which corresponds to a distance of 1.86 cm from the cells (Figure 2C), as stated by the manufacturer (IBIDI GmbH, 82152 Martinriesd, Munich, Germany). PBM therapy was administered through the powers of 0.25, 0.50, 0.75, 1.00, or 1.25 W treated in continuous wave (CW) mode with an exposure time of 60 s and a spot size of 1 cm^2^. 0 W denotes the untreated controls. To perform blinded treatments, a 635 nm red-light guide (negligible power, <0.5 mW) with the laser device switched to silent mode was used (Figure 2C). As the culture medium may influence the precise dose delivery, treatments were performed in the presence of only 10 μL; (of 1 mL) medium to avoid cell dehydration during the 60 s of treatment. To avoid beam reflection, the multiwell were placed on an absorbing material. The precision of the laser dose was confirmed with a power meter (Pronto-250, Gentec Electro-Optics, Inc. Québec, QC G2E 5N7, Canada).

Adverse events due to possible thermal effects were avoided by monitoring the treatments with a thermal camera (FLIR ONE Pro-iOS, FLIR Systems, Inc. designs, Portland, USA, dynamic range: −20 °C/+400 °C; resolution 0.1 °C). To avoid bias, the cell growth, PBM treatments, and cell analyses were performed by different operators to ensure blinding. After treatments, cells samples were then processed to evaluate the variation of the energetic metabolism (oxygen consumption, ATP synthesis, OxPhos efficiency and lactic acid production (Figure 2D–F)), oxidative stress (lipid peroxidation; Figure 2F), antioxidant enzyme activity (glutathione reductase and catalase; Figure 2F), markers regulating apoptosis (Figure 2G), cell cycle, and viability (Figure 2H,I), as described below.

### 2.2. Cell Lines and Treatments

As a Human Head and Neck Squamous Cell Carcinoma (HNSCC) model, the OHSU-974 cell line was employed. Since this cell line was isolated from Fanconi Anemia Type A Patients, to avoid any bias associated with the mutation of the FANC-A gene, the cell line has been corrected with S11FAIN retrovirus (OHSU-974 FAcorr). The cell line was obtained from the “Cell Line and DNA Biobank from patients affected by Genetic Diseases” G. Gaslini Institute–Telethon Genetic Biobank Network, Genoa, Italy.

OHSU-974 FAcorr was grown in RPMI supplemented with 10% fetal calf serum and antibiotics at 37 °C with 5% CO_2_. PBM treatments were performed as described (2.01 above), media was replenished, and cells were maintained in culture for 24, 48, and 72 h. Cells were harvested and used for protein or biochemical assays. 

### 2.3. F_o_-F_1_ ATP Synthase Activity Assay

The F_o_-F_1_ ATP synthase (ATP Synthase) activity was evaluated on 20,000 cells permeabilized with 0.03 mg/mL digitonin for 1 min. Cells were incubated for 10 min in a medium containing: 50 mM Tris-HCl pH 7.4, 50 mM KCl, 1 mM EGTA, 2 mM MgCl_2_, 0.6 mM ouabain, 0.25 mM di(adenosine)-5-penta-phosphate (Ap5A, adenylate kinase inhibitor), and 25 μg/mL ampicillin (0.1 mL final volume). ATP synthesis was induced by the addition of 10 mM pyruvate plus 5 mM malate, and 0.1 mM ADP to stimulate the pathway composed by Complexes I, III, and IV. The reaction was monitored for 2 min, every 30 s, with a luminometer (GloMax^®^ 20/20n Luminometer, Promega Italia), by the luciferin/luciferase chemiluminescent method, with ATP standard solutions between 10^−8^ and 10^−5^ M (luciferin/luciferase ATP bioluminescence assay kit CLS II, Roche, Basel, Switzerland). Data were expressed as nmol ATP/min/10^6^ cells [5].

### 2.4. Oxygen Consumption Assay 

Oxygen consumption was measured at 25 °C in a closed chamber using an amperometric electrode (Unisense Microrespiration, Unisense A/S). Cells were permeabilized with 0.03 mg/mL digitonin for 1 min, centrifuged for 9 min at 1000 rpm, and resuspended in phosphate buffer saline (PBS). The same solution was used in the oximetry measurements. A total of 20,000 cells were used for each experiment. Moreover, 10 mM pyruvate plus 5 mM malate plus 0.1 mM ADP were added to stimulate the pathway composed by Complexes I, III, and IV. Data were expressed as nmol O/min/10^6^ cells. 

The oxidative phosphorylation (OxPhos) efficiency (P/O ratio) was calculated as the ratio between the concentration of the produced ATP and the amount of consumed oxygen in the presence of respiring substrates and ADP [5]. In efficient mitochondria, the P/O value, in the presence of pyruvate and malate as respiring substrates, is around 2.5, indicating that the oxygen consumption can be completely devoted to energy production [46]. Conversely, when the P/O value is less than 2.5, part of the oxygen is not used for energy production, but concurs with the formation of reactive oxygen species (ROS) [47].

### 2.5. Lactate Release Assay

Lactate concentration was spectrophotometrically assayed in the growth medium, following the reduction of NAD^+^ at 340 nm. The assay medium contained 100 mM Tris-HCl, pH 8, and 5 mM NAD^+^. Samples were spectrophotometrically analyzed before and after the addition of 4 μg of purified lactate dehydrogenase. Data were normalized based on cell numbers [48].

### 2.6. Cell Homogenate Preparation 

Cultured cells were centrifuged at 1000 rpm for 5 min to remove the growth medium. The pellet was washed in PBS, resuspended in Milli-Q water, and sonicated twice for 10 s in ice, with a 30 s break to prevent mixture warming, using the Microson XL Model DU-2000 (Misonix Inc. Farmingdale, NY, USA). Total protein content was estimated using the Bradford method [49].

### 2.7. Evaluation of Malondialdehyde 

To assess lipid peroxidation, malondialdehyde (MDA) concentration was evaluated using the thiobarbituric acid reactive substances (TBARS) assay. This test is based on the reaction of MDA, a breakdown product of lipid peroxides, with thiobarbituric acid (TBA). The TBARS solution contained 15% trichloroacetic acid (TCA) in 0.25 N HCl and 26 mM thiobarbituric acid. To evaluate the basal concentration of MDA, 600 μL of TBARS solution were added to 50 μg of total protein dissolved in 300 μL of Milli-Q water. The mix was incubated for 60 min at 95 °C. Afterwards, the sample was centrifuged at 14,000 rpm for 2 min and the supernatant was spectrophotometrically analyzed at 532 nm [50].

### 2.8. Enzymatic Antioxidant Defenses Assay

Glutathione reductase activity was spectrophotometrically assayed, following the oxidation of NADPH at 340 nm. The assay mix contained 100 mM Tris HCl, pH 7.4, 1 mM EDTA, 5 mM GSSH, and 0.2 mM NADPH [51]. Catalase activity was spectrophotometrically assayed, following the decomposition of H_2_O_2_ at 240 nm. The assay mix contained 50 mM phosphate buffer, pH 7.0, and 5 mM H_2_O_2_ [48]. For both assays, 20 μg of the total protein was used, and data were normalized on the sample protein content.

### 2.9. Western Blot (WB) Analysis 

Denaturing electrophoresis (SDS-PAGE) was performed on 4–20% gradient gels (BioRad). In total, 30 μg of proteins were loaded for each sample. The following antibodies were employed: anti-Bcl2 (Cell Signaling, #15071), anti-BAX (MilliporeSigma, cod: 06–449), anti-phospho-p53 (Ser 15) (Santa Cruz Biotechnology, cod: sc-101762), anti-p53 (Santa Cruz Biotechnology, cod: sc-98), and anti-Actin (Santa Cruz Biotechnology, cod: sc-1616). All primary antibodies were diluted 1:1000 in PBS plus 0.15% tween (PBST). Specific secondary antibodies were employed (Sigma-Aldrich), diluted 1:10,000 in PBST. Bands were detected and analyzed for optical density using an enhanced chemiluminescence substrate (ECL, BioRad), a chemiluminescence system (Alliance 6.7 WL 20M, UVITEC), and UV1D software (UVITEC). Bands of interest were normalized for actin levels in the same membrane.

### 2.10. Evaluation of Cell Cycle and Cellular Activation 

Flow-cytometric analysis of DNA content was performed on cells permeabilized with 0.05% Triton X-100 and stained with 30 mg/mL PI plus 0.5 mg/mL RNase for 30 min. Samples were measured on a FACS-Calibur flow cytometer (Becton Dickinson) and cell cycle phase distributions were achieved from the DNA content histograms of at least 10,000 cells [48]. To evaluate the cellular grow rate, OHSU-974 FAcorr were counted by a Burker chamber after trypan blue staining.

### 2.11. Statistical Analysis

Data were analyzed using a one-way or two-way ANOVA, as appropriate, followed by Tukey’s or Sidak’s multiple comparison test, respectively, using Prism 8 Software. Data are expressed as mean ± standard deviation (SD) and are representative of at least three independent experiments. An error with probability *p* < 0.05 was considered statistically significant.

## 3. Results

### 3.1. Cancer Cell Mitochondrial Metabolism Is Modulated by PBM Dose 

**Figure 3:**
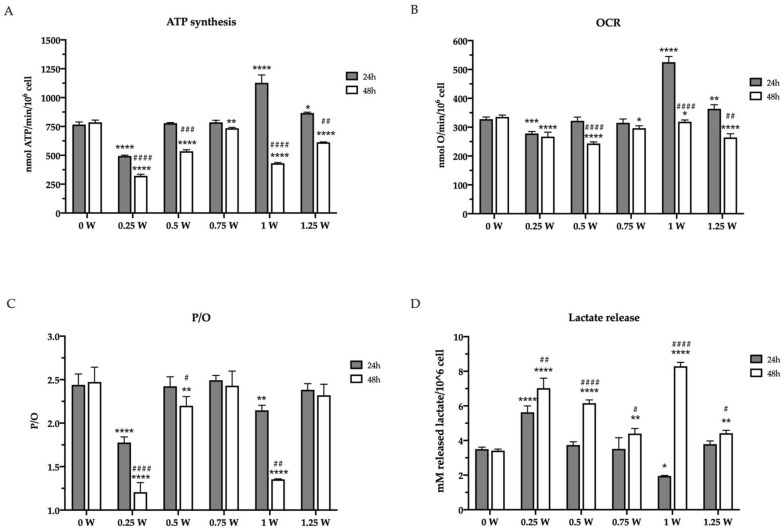
Glucose metabolism modulation in OHSU-974 FAcorr cells after 808-nm laser irradiation. (**A**) ATP synthesis through FoF1-ATP synthase; (**B**) the oxygen consumption rate (OCR). For both graphs, data are obtained in the presence of pyruvate plus malate as respiring substrates. (**C**) P/O value, calculated as the ratio between the synthesized ATP and the consumed oxygen. (**D**) Lactate release in the growth medium. In each graph, grey and white columns represent the data obtained 24 or 48 h after the laser irradiation, respectively. Data are reported as mean ± SD, and each graph is representative of at least three independent experiments. Statistical significance was tested by a two-way ANOVA. *, **, ***, and **** represent *p* < 0.05, 0.01, 0.001, and 0.0001, respectively, between the treated sample and the untreated sample (0 W) used as control, at the same time after irradiation. #, ##, ###, and #### represent *p* < 0.05, 0.01, 0.001, and 0.0001, respectively, between the values obtained after 24 h and 48 h from the laser irradiation in the sample treated with the same laser power.

The effects of an 808 nm laser on OHSU-974 mitochondrial energy metabolism were evaluated in terms of ATP synthesis and oxygen consumption rate (OCR) after 24 and 48 h from irradiation at different powers (0.25–1.25 W). The results, reported in Figure 3, show that, 24 h after the irradiation, only 0.25 W and 1 W affected the ATP synthesis, inducing a decrement of about 30% and an increment of 35%, respectively. By contrast, 48 h after the laser treatment, all the powers, except the 0.75 W, determined a significant impairment of energy production compared with the untreated sample (0 W) (Figure 3A). A similar trend, although less evident, was observed with OCR (Figure 3B), suggesting a loss in mitochondrial energy production. Interestingly, in some cases (0.25, 0.5, and 1 W), the reduction of mitochondrial aerobic activity is also associated with an impairment of the OxPhos efficiency, as indicated by the P/O ratio value (Figure 3C). Specifically, 0.25 W and 1 W treatment determined a severe P/O ratio decrement already 24 h after the irradiation, which worsened further at 48 h, while 0.5 W induced a slight decrease only at 48 h. These observed modulations of mitochondrial energetics suggest PBM treatments reorganize glucose metabolism in OHSU-974 FAcorr. 

The mitochondrial metabolism modulation observed after the laser irradiation determined a reorganization of the glucose metabolism. For example, after the first 24 h, the cells treated with 1 W showed a decrement of lactate release, a sign of the anaerobic glycolysis reduction, probably due to the enhancement of OxPhos activity. Conversely, the treatment at 0.25 W induced the opposite effect, incrementing the lactate release (Figure 3D). This parallelism has also been maintained after 48 h from irradiation, as all samples displayed an increment of the released lactate in accordance with the aerobic metabolism reduction.

### 3.2. PBM Treatments Modulate Redox Status in Cancer Cells 

**Figure 4:**
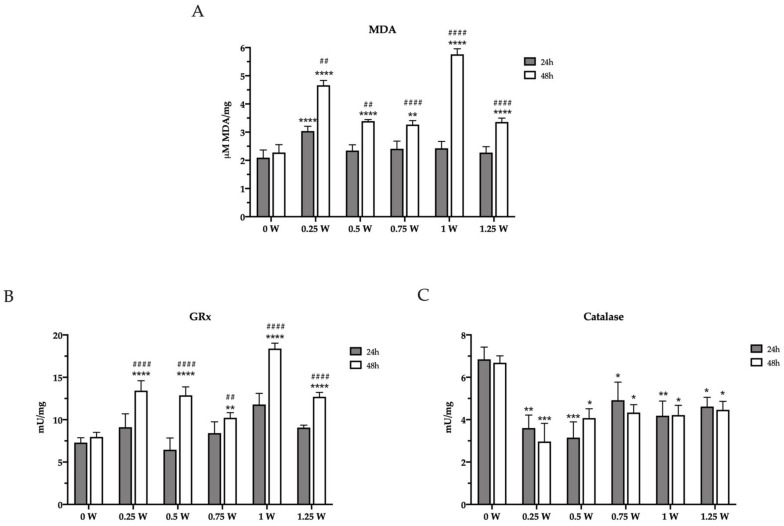
Lipid peroxidation and modulation of enzymatic antioxidant defenses in OHSU-974 FAcorr cells after 808-nm laser irradiation. (**A**) Malondialdheyde (MDA) level. (**B**) Glutathione reductase (GRx) activity. (**C**) Catalase activity. In each graph, grey and white columns represent the data obtained 24 or 48 h after the laser irradiation. Data are reported as mean ± SD, and each graph is representative of at least three independent experiments. Statistical significance was tested by a two-way ANOVA. *, **, *** and **** represent *p* < 0.05, 0.01, 0.001 and 0.0001, respectively, between the treated sample and the untreated sample (0 W) used as control, at the same time after irradiation. ## and #### represent *p* < 0.05, and 0.0001, respectively, between the values obtained after 24 h and 48 h from the laser irradiation in the sample treated with the same laser power.

Since altered mitochondrial metabolism is associated with increased oxidative stress, the concentration of malondialdehyde (MDA) has been evaluated as an end-point of oxidative damages. After the first 24 h elapsed from irradiation, only 0.25 W determined an increment in MDA level with respect to the control (0 W), according to the reduced mitochondria activity and P/O ratio. Conversely, the MDA level was higher in all treated samples compared to the untreated sample, after 48 h from the laser treatment, reaching the maximum level in the 1 W sample. (Figure 4A). Since the increment of oxidative stress is normally counteracted by the endogenous antioxidant defenses, the activity of glutathione reductase (GRx) and catalase were assayed. As expected, GRx activity increased 48 h after PBM treatments (Figure 4B). These changes followed a similar trend as MDA levels, probably as an attempt to balance the enhancement of the oxidative stress. By contrast, surprisingly, PBM treatment determined a catalase activity reduction in all treated samples as early as 24 h after irradiation that was sustained over 48 h (Figure 4C). Overall, these changes in the redox status in cancer cells following PBM treatments aligned well with the observed altered mitochondrial bioenergetics. 

### 3.3. PBM Treatments Decreased Cancer Cell Viability 

**Figure 5:**
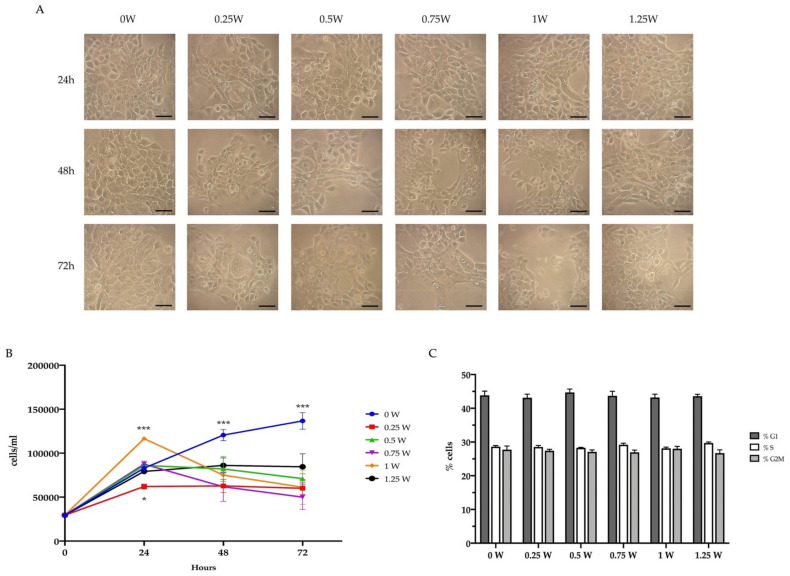
Effect of 808-nm laser irradiation on OHSU-974 FAcorr vitality and proliferation. (**A**) Example of OHSU-974 FAcorr morphology observed at the transmitted-light microscope. Scale bar corresponds to 100 μm. Each panel is representative of at least five fields of three independent experiments. (**B**) Growth curve of OHSU-974 FAcorr treated or not with an 808-nm laser. (**C**) Proliferative response of OHSU-974 FAcorr cells treated or not with an 808-nm laser 48 h after the irradiation. For panels B and C, data are reported as mean ± SD, and each graph is representative of at least three independent experiments. Statistical significance was tested by two-way ANOVA. * and *** represent *p* < 0.05 and 0.001, respectively, between the treated sample and the untreated sample (0 W) used as control, at the same time after irradiation. No significant differences were observed in Panel C.

In addition to altering glucose metabolism, PBM treatments affected the OHSU-974 FAcorr viability. In detail, 24 h after the irradiation, 0.25 and 1 W determined a decrement and an increment in the cell number, respectively, compared to the control and the other treated samples (Figure 5A,B). However, after 48 and 72 h from the laser irradiation, all treated samples showed a significant decrement in the cell number compared to the control (Figure 5A,B). Moreover, cell morphology analysis showed the presence of several apoptotic bodies (Figure 5A). However, cell cycle analysis noted a similar pattern, indicating surviving cancer cells were not dormant or growth-arrested (Figure 5C). 

### 3.4. 808-nm Laser Irradiation Modulate the Activation of p53 and the Protein Expression of Bcl2 and Bax

**Figure 6:**
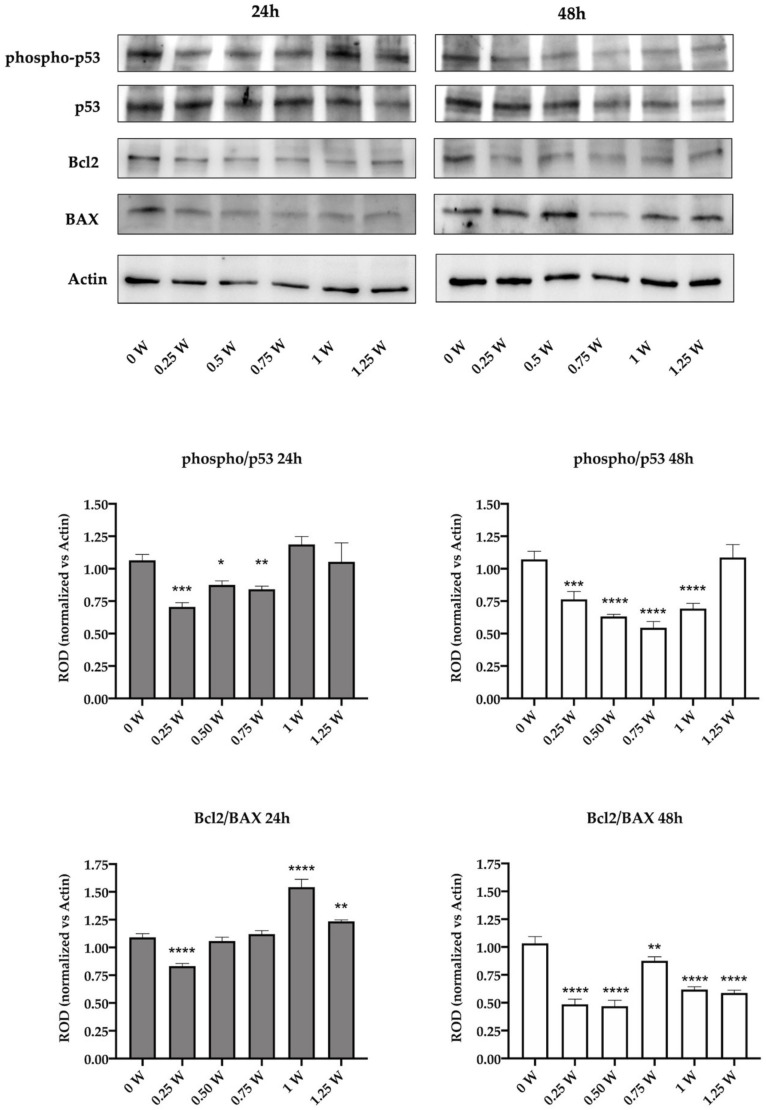
Effect of 808-nm laser irradiation on p53 activation and the Bcl2/Bax ratio. The upper part of the figure shows the western blot (WB) signals of the expression of phospho-p53, p53, Bcl2, and Bax in the OHSU-974 FAcorr 24 or 48 h after treatment with the 808-nm laser at powers ranging from 0 to 1.25 W. The graphs below show the ratio between the expression of phospho-p53 (active form) and p53 and the ratio between Bcl2 and Bax (anti-apoptotic/pro-apoptotic). WB signals are representative of at least three independent experiments. For the graphs, data are reported as mean ± SD, and each graph is representative of at least three independent experiments. Statistical significance was tested by a one-way ANOVA. *, **, ***, and **** represent a *p* < 0.05, 0.01, 0.001, and 0.0001, respectively, between the treated sample and the untreated sample (0 W) used as control, at the same time after irradiation.

To confirm the activation of the apoptotic process in the lasered OHSU-974 FAcorr cells, the expression of phospho-p53, p53, Bcl2, and Bax has been evaluated, by western blot. Data show that in the first 24 h after the laser irradiation, the p53 phosphorylation appeared impaired only in 0.25, 0.5, and 0.75 W cells, while other samples displayed an expression level similar to that of the control. Conversely, after 48 h of laser irradiation, all samples except the one treated at 1.25 W showed the impairment in the activation of p53, suggesting a minor ability to check the DNA damage (Figure 6). The evaluation of the expression of anti-apoptotic Bcl2, the pro-apoptotic Bax, and their ratio show that at 24 h after the laser treatment only in 0.25 W sample the equilibrium was unbalanced towards the apoptosis activation. Conversely, 1 W and 1.25 W seem to have a diametral effect, increasing the Bcl2/Bax ratio. Forty-eight hours after the irradiation, all the treated samples show a sharp decrement of the Bcl2/Bax ratio, suggesting an activation of the apoptotic pathway (Figure 6), and confirming the morphological data reported in Figure 5.

## 4. Discussion

The effect of PBM on cellular energetic metabolism was described [1,8]. Mitochondria play a pivotal role in this light therapy through the modulation of ATP and ROS production [3,4,5,6]. Because the metabolic activity is altered in cancerous cells relative to normal cells [52,53], the safeness of PBM therapy in the treatment of radio- and chemo-therapy side effects was, for many years, supposed. Basically, cancer reprograms the cell metabolic pathway, which starts to constitutively take up glucose and produce lactate regardless of oxygen availability [52,54]. This process is noted as the Warburg effect or aerobic glycolysis [55]. Therefore, the effect of PBM on mitochondria activity followed by ATP production increment would stimulate cancer cells apoptosis [31,36]. Nevertheless, *in vitro,* and in vivo studies showed conflicting results concerning the effects of PBM on the proliferation of cancer [23,24,31,35,56,57,58,59]. Recently, the role of aerobic glycolysis supported by oncogenes was confirmed, but studies have also demonstrated that the tumor cells prevalently produce energy through glucose oxidation and oxidative phosphorylation, which takes place in mitochondria [52,54]. Therefore, despite cancer cells having high glycolytic rates, main ATP generation would occur through mitochondrial function, apart from the tumors bearing mutations in mitochondrial respiration proteins [52,60,61]. Thus, the effects of PBM on cancer cell metabolism should be carefully investigated to support the therapy as safe [38].

Our data showed that 808-nm wavelength PBM at different powers and fluences interact with mitochondrial energetic metabolism of OHSU-974 FAcorr, as previously observed in normal cells [1,2,12,26,41]. However, the aerobic mitochondria activity was inhibited, except samples irradiated with 1 W (60 s, 1 cm^2^), which, 24 h after treatment, drastically increased the mitochondria activity. However, 48 h after the irradiation the sample experienced a drastic decrement in oxygen consumption and ATP synthesis, in line with the effect of the other parameters. Basically, 48 h after the irradiation, the 808-nm laser light led to mitochondria uncoupling with the exclusion of 0.75 and 1.25 W (60 s, 1 cm^2^). This means that the treatment with an 808-nm laser not only determined a decrease of the mitochondrial energy function but also a partial OxPhos inefficiency, causing a further reduction of the energy production and an increment of oxidative stress production. Interestingly, the effect seems to follow the window effect previously described for the interaction of 980-nm wavelength with isolated-mitochondria of mammals [5]. 

More in general, since the cancer cells are characterized by the Warburg effect, which consists of a metabolic switch from aerobic metabolism to anaerobic glycolysis, resulting in a sharp increase in glucose consumption [55], the impairment of mitochondria metabolism should not cause excessive imbalances as these cells. However, this metabolic view has recently been revised, as mitochondria, in several in vivo and in vitro tumor models, appear to play a pivotal role in cancer proliferation and aggressiveness. This apparent discrepancy could be explained since the high consumed glucose, in the aerobic condition, is used both to sustain the anaerobic glycolysis and to provide the necessary building blocks for an accelerated proliferation by other metabolic pathways [34,62]. This new vision is in accordance with the increment of lactate release observed after 48 h from the laser irradiation. In fact, this metabolic switch could be considered both an attempt to compensate for the energy production and a system recycling the reduced coenzymes, NADH *in primis*, to maintain active energy metabolism. [52,62].

The increase of mitochondria respiratory chain activity and even more mitochondria unbalance show the unphysiological implementation of ROS production [63]. Note that mitochondria are one of the most prominent sources of reactive oxygen species and contribute to oxidative stress [63,64]. High levels of ROS may drown out the responsive antioxidant machinery of cancer cells, leading to their death, but moderate levels of ROS, particularly H_2_O_2_, can oxidize the cysteine residues of proteins and support cancer cell development, proliferation, and adaptation [50,64,65]. Consequently, the influence of ROS on cellular homeostasis is complex, showing both anti- and pro-tumorigenic effects [66].

OHSU-974 FAcorr cells irradiated through 808-nm experienced oxidative stress, as pointed out by the increment of glutathione reductase activity. This enzyme plays a role in the pathway involved to maintain the redox balance of cancer cells under their pro-oxidative environmental conditions and provides survival support for tumors against chemotherapeutic drugs and radiotherapy [67,68,69]. However, its contribution seems not sufficient to counteract the stress generated by 808-nm PBM. In fact, lipid peroxidation is higher in all the OHSU-974 FAcorr cell samples than in the no-irradiated control. 

Since, mitochondrial ROS modulation control pro- or anti-tumorigenic cell signaling [67], the efficient ability of cancer cells to develop mechanisms keeping the increased oxidative stress in check and promoting resistance to apoptosis was extensively described [66,67,68,69]. Therefore, the influence of 808-nm PBM on the pro- and anti-oxidative equilibrium in the cancer cell is of interest. The effectiveness of cancer cells’ antioxidant machinery is also guaranteed through the activity of the catalase enzyme [70]. Indeed, catalases are ubiquitous heme-enzymes preventing oxidative damage by degrading hydrogen peroxide to oxygen and water with high efficiency [71]. Catalase showed a preventive role against a high-level of pro-apoptotic oxidative stress in cancer cells [52,70,71]. Moreover, because oxygenated and deoxygenated hemoglobin absorb light equally at 800 nm [72,73] the catalase heme-group may be a target of 808-nm PBM. Our data showed OHSU-974 FAcorr cells significantly decrease catalase activity after irradiation through all the laser therapies (0.25, 0.50, 0.75, 1.00, and 1.25 W; 60 s, 1 cm^2^). Therefore, regardless of the effect of laser light therapy on mitochondria activity (decrease, increase, or uncoupling), the OHSU-974 FAcorr cells seem not able to counteract the oxidative stress generated, despite the enhancement of GRx activity, probably because of a severe inhibition of catalase activity by the 808-nm PBM. 

The 808-nm laser treatment on OHSU-974 FAcorr also determines the alterations in the vitality rate. Our data, in fact, show that 24 h after the irradiation, the cell number decreased in the sample treated with 0.25 W and increases with 1 W treatment, following the modulation of the energy metabolism. However, 48 h and 72 h after the irradiation, a decrement in the cellular viability and an increment of apoptotic bodies were observed. These data are confirmed by the expression of two apoptosis markers, Bcl2 and Bax, anti- and pro-apoptotic proteins, respectively. As reported by the WB signals, the ratio between Bcl2 and Bax drastically decreased after 48 from the laser treatment, suggesting an imbalance towards the activation of apoptosis, which could be responsible for the cell number reduction. Moreover, the p53 pathway activation also appeared inhibited, indicating that the treated cells were less able to verify the DNA quality and to manage possible errors.

Given the heterogeneity of the experimental setup, laser parameters, and results, a comparison between our data and literature has no consistent conclusions. However, our data seem to follow the preliminary results on oral squamous cell carcinoma (SCC) cell line of Gonçalves De Faria and collaborators [38], which through a wavelength of 780-nm and 3.4 J/cm^2^, observed that “PBM decreases the redox state of oral cancer by possibly increasing glycolysis and affects normal and tumor cells through distinct pathways”. Additionally, human cervical cancer cells (HeLa cell line) experienced an 808-nm PBM induced pro-apoptotic effect dependent on irradiation dose [74]. The more evident apoptosis rate was noted for the lower irradiation doses, 0.3 J/cm^2^ and 3 J/cm^2^, than the highest, 30 J/cm^2^. 

The 800-nm range of wavelengths showed also a significant inhibitory effect on the proliferation of SCC cells originated from the tongue (810-nm, 4 J/cm^2^) [75] and SCC from the gingival mucosa (805 nm, 20 J/cm^2^ [76] and 805-nm, 2–20 J/cm^2^ [77]). Conversely, cellular proliferation or activation was positively stimulated in epithelial tumor cells from laryngeal carcinoma (809-nm; 1.96, 3.92, and 7.84 J/cm^2^) [78], human osteosarcoma cell line, SAOS−2, (830-nm, from 1.7 to 25.1 J/cm^2^) [79], SCC of the larynx, H.Ep.2 cells, (830-nm, 4 J/cm^2^) [80], and epidermoid carcinoma (KB) cells (830-nm, 4 J/cm^2^) [81].

## 5. Conclusions

In conclusion, our data met the primary and secondary endpoints. Indeed, despite the limitations of the in vitro experimental setup, our results consistently showed the harmful effect of 808-nm PBM on the OHSU-974 FAcorr. The ability of 808-nm wavelength laser light to interact with cell photo-acceptors localized into the mitochondria respiratory chain and the catalase enzyme negatively impacts carcinoma cell homeostasis. This action-reaction effect would affect the energetic metabolism equilibrium between the mitochondrial oxidative phosphorylation and glycolysis followed by lactic acid fermentation, as well as the antioxidant enzymes chain of cancer cells. Therefore, the OHSU-974 FAcorr cells would fail to counteract the increasing ROS production, which would stimulate the pro-apoptotic cellular pathways. Despite the promising results, pre-clinical and clinical studies are necessary to confirm the PBM safeness as supportive therapy for toxicities related to anti-cancer treatment and better investigate its possible anti-cancer coadjuvant therapy use.

## Data Availability

Data available on request from the authors.
